# Long non‐coding RNA highly up‐regulated in liver cancer promotes epithelial‐to‐mesenchymal transition process in oral squamous cell carcinoma

**DOI:** 10.1111/jcmm.14160

**Published:** 2019-01-24

**Authors:** Wen Su, Jing Tang, Yufan Wang, Shuai Sun, Yuehong Shen, Hongyu Yang

**Affiliations:** ^1^ Department of Oral and Maxillofacial Surgery Peking University Shenzhen Hospital Shenzhen Guangdong China; ^2^ Peking University Shenzhen Hospital Clinical College, Anhui Medical University Hefei Anhui China; ^3^ Jingzhou Central Hospital Jingzhou Hubei

**Keywords:** epithelial‐to‐mesenchymal transition, highly up‐regulated in liver cancer, long non‐coding RNA, oral squamous cell carcinoma

## Abstract

Oral squamous cell carcinoma (OSCC) is an oral and maxillofacial malignancy that exhibits high incidence worldwide. In diverse human cancers, the long non‐coding RNA (lncRNA) highly up‐regulated in liver cancer (HULC) is aberrantly expressed, but how HULC affects OSCC development and progression has remained mostly unknown. We report that HULC was abnormally up‐regulated in oral cancer tissues and OSCC cell lines, and that suppression of HULC expression in OSCC cells not only inhibited the proliferation, drug tolerance, migration and invasion of the cancer cells, but also increased their apoptosis rate. Notably, in a mouse xenograft model, HULC depletion reduced tumorigenicity and inhibited the epithelial‐to‐mesenchymal transition process. Collectively, our findings reveal a crucial role of the lncRNA HULC in regulating oral cancer carcinogenesis and tumour progression, and thus suggest that HULC could serve as a novel therapeutic target for OSCC.

## 1 INTRODUCTION

Oral squamous cell carcinoma (OSCC), the most common malignancy of the head and neck, accounts for roughly 3% of all cancers, and more than half a million patients worldwide are diagnosed with oral cancer every year.^1^, ^2^ In approximately one‐third of the patients, the cancer is detected at an early stage, and the survival rate of these patients is 90%.^2^ However, the 5‐year survival rate of patients with oral cancer has remained at nearly 50%, and the rate is even lower in the case of patients with lymph node metastasis and distant metastases.^3^, ^4^, ^5^ OSCC occurrence and development are recognized to be complex processes, involving numerous genes and non‐coding RNAs, but the mechanism of OSCC initiation and progression remains unclear.

Several recent studies have revealed that long non‐coding RNAs (lncRNAs) play a crucial role in OSCC.^6^, ^7^, ^8^, ^9^ LncRNAs are >200‐nt‐long endogenous RNAs that neither contain a notable open reading frame nor encode proteins, and lncRNAs participate in the regulation of tumour gene expression, protein localization and other physiological processes.^10^, ^11^, ^12^, ^13^ The lncRNA highly up‐regulated in liver cancer (HULC), which was first reported to be abundantly expressed in primary liver tumours, is ~1600 nt long and contains two exons that are not translated into proteins.^14^ Transcription of HULC yields ~500 nt long, spliced and polyadenylated ncRNA that localizes to the cytoplasm, where it has been reported to be associated with ribosomes.^15^ HULC has been shown to perform critical functions in diverse tumours, including gastric cancer, pancreatic cancer, osteosarcoma and liver metastasis of colorectal cancer.^14^, ^16^, ^17^, ^18^, ^19^ However, no study to date has reported a regulatory role of HULC in OSCC.

To investigate HULC function in OSCC development, we quantified HULC expression levels in oral cancer tissues and adjacent normal tissues by using qRT‐PCR. HULC expression was higher in the cancer tissues than in the normal tissues, and, similarly, was higher in OSCC cell lines than in normal keratinocytes, and HULC down‐regulation in the OSCC cell lines SCC15 and SCC25 affected the proliferation, migration and invasion abilities of these cells. Moreover, in a nude mouse xenograft model that we constructed, HULC depletion reduced tumorigenicity and inhibited the epithelial‐to‐mesenchymal transition (EMT) process. Our results not only reveal a previously unreported regulatory role of HULC in OSCC, but also suggest that HULC represents a potential therapeutic target for OSCC.

## 2 MATERIALS AND METHODS

### 2.1 Patients and tissue samples

Oral cancer tissues and their adjacent normal tissues were obtained from 30 oral cancer patients at the Department of Oral and Maxillofacial Surgery, Peking University Shenzhen Hospital (Shenzhen, China), between 2017 and 2018. None of the patients had received chemotherapy or radiotherapy before surgery. The tissue specimens were snap frozen in liquid nitrogen immediately after resection and stored at −80°C until RNA extraction. Oral cancer was diagnosed and classified through pathological examination based on the World Health Organization classification system. Informed consent was obtained from all patients in accordance with the ethical guidelines of Peking University (Protocol No. 37923/2‐3‐2012). This study was approved by Ethics Committee of Peking University Health Science Center (IRB00001053‐08043).

### 2.2 Cell culture and transfection

The human OSCC cell lines SCC9, SCC15, SCC25 and CAL27 were obtained from the College of Stomatology, Wuhan University (Wuhan, China). HOK cells were obtained from the cell bank of the Chinese Academy of Sciences (Shanghai, China). All cells were cultured at 37°C and 5% CO_2 _in a humidified atmosphere. SCC15, SCC25, CAL27 and HOK cells were cultured in Dulbecco's modified Eagle medium (DMEM; Gibco) containing 10% foetal bovine serum (FBS; Gibco) and 1% penicillin/streptomycin (Life Technologies Inc). SCC9 cells were cultured in DMEM/Ham's F12 medium (1:1) containing 10% FBS and 1% penicillin/streptomycin. Plasmid and siRNA transfections were conducted using Lipofectamine 3000 (Invitrogen).

Two HULC siRNAs were synthesized by RiboBio (Guangzhou, China): HULC siRNA‐1:5′‐CCGGAAUAUUCUUUGUUUAUU‐3′; and HULC siRNA‐2:5′‐UAAACAAAGAAUAUUCCGGUU‐3′. Both siRNAs yielded identical results, and the results obtained with HULC siRNA‐1 are presented here. Two non‐targeting siRNAs were used as negative controls: control siRNA‐1:5′‐CCUUAUAUGUUCUGGAAUUUU‐3′; and control siRNA‐2:5′‐UAAAACGAAUGGAAUUCACUU‐3′. The results shown here were obtained using control siRNA‐1. For HULC shRNA construction, a lentiviral vector was obtained from GenePharma (Shanghai, China), and the primers for the HULC shRNA were the following: forward, 5′‐GGAGAACACTTAAATAAGTTT‐3′; reverse, 5′‐ACTTATTTAAGTGTTCTCCTA‐3′.

### 2.3 RNA extraction and qRT‐PCR

RNA extraction and qRT‐PCR were performed as previously described.^20^ Total RNA from frozen tissues or cultured cells was extracted using TRIzol reagent (Invitrogen), according to the manufacturer's protocol. A PrimeScript RT Reagent Kit (Takara Bio, Nojihigashi, Kusatsu, Japan) was used for reverse‐transcribing the RNA into cDNA, as per the manufacturer's instructions. qRT‐PCR was performed with SYBR‐Green Premix Ex Taq (Takara Bio) and was monitored using an ABI PRISM 7500 Sequence Detection System (Applied Biosystems, Life Technologies). Comparative quantification was performed with either the ΔCt or the 2^–ΔΔCt^ method (as indicated in the figure). The following primers were used in the qRT‐PCR assays: HULC: forward, 5′‐TCATGATGGAATTGGAGCCTT‐3′; reverse, 5′‐CTCTTCCTGGCTTGCAGATTG‐3′; GAPDH: forward, 5′‐CAGCCAGGAGAAATCAAACAG‐3′; reverse, 5′‐GACTGAGTACCTGAACCGGC‐3′.

### 2.4 CCK‐8 assay

We seeded 100 μl of transfected OSCC (SCC15 and SCC25) cell suspensions in 96‐well plates at a density of 2 × 10^3^ cells/well, and then added CCK‐8 solution (Beyotime, Shanghai, China) at 10 μl/well at 24, 48, 72 and 96 hours. The plates were incubated for 1 hour and the 450‐nm absorbance was measured, and the OD values at various time‐points were compared.

### 2.5 EdU cell‐proliferation assay

Transfected OSCC cells were seeded in 96‐well plates at a density of 4 × 10^4^ cells/well and cultured to logarithmic growth phase. The cells were incubated with diluted EdU solution for 2 hours, fixed with 4% paraformaldehyde, and then stained with Apollo staining‐reaction solution and Hoechst 33342 reaction solution in the dark. Subsequently, images were acquired and analysed.

### 2.6 Flow cytometry

Cells were routinely transfected and cultured for 48 hours and then digested with trypsin without EDTA. An Annexin V‐FITC apoptosis assay kit (Biyuntian Biotechnology Co., Ltd.) was used to estimate the apoptosis rate, according to the manufacturer's instructions. Cells were suspended in 1× annexin‐binding buffer, and then 5 μl of Annexin V and 1 μl of PI reagents were added to 100 μl of the cell suspension and mixed. The mixture was incubated in the dark for 15 minutes at room temperature, and then 400 μl of the 1× annexin‐binding buffer was added to each sample to terminate the staining. The apoptosis rate was determined using a FACSCalibur flow cytometer (BD).

### 2.7 Hoechst staining assay

We plated 4 × 10^5^ cells/well in 24‐well plates containing sterile glass coverslips, and following overnight incubation, fixed the cells with 4% paraformaldehyde for 15 minutes. Hoechst 33258 staining solution (Beyotime) was added and the cells were incubated for 10‐15 minutes, and after air‐drying, images were acquired and examined for typical apoptosis‐related changes (chromatin concentration, aggregation, destruction, etc.).

### 2.8 Wound‐healing assay

Transfected cells were spread on 6‐well plates and cultured until confluence. Before wounding, the cells were cultured in DMEM without FBS for 1 day. A sterile 200‐μl pipette tip was used to scratch the cell monolayers, and after wounding, the cells were washed thrice with phosphate‐buffered saline (PBS) and incubated with fresh medium containing 10% FBS. Images were acquired at 0 and 48 hours or 72 hours.

### 2.9 Migration and invasion assays

Migration and invasion assays were performed with , respectively, Transwell chambers and Matrigel pre‐coated Transwell chambers (Corning, NY). Cells were resuspended in DMEM without FBS and added to the upper chamber, and medium containing 10% FBS was added to the lower chamber; after incubation for 24 or 48 hours, the cells in upper chamber were wiped off, and the cells in the lower chamber were fixed in 4% paraformaldehyde, stained with 0.1% crystal violet, washed with PBS, and dried. Lastly, images were acquired and analysed.

### 2.10 Cell‐viability assay

The cell‐viability assay was designed according to the method of Wang et al.^21^ CDDP (≥98% pure; Dalian Meilun Biotech Co., Ltd., Dalian, China) was dissolved in 0.1% DMSO. Cells were seeded into 96‐well plates and treated with different doses of CDDP (0, 0.156, 0.312, 0.625, 1.25, 2.5, 5, 10, 20 μg/ml) for 72 hours, after which cell viability was assayed by using the MTS method with a CellTiter 96® AQueous One Solution Cell Proliferation Assay Kit (Promega Corporation, Madison, WI) according to the manufacturer's instructions. After 4‐hours incubation at 37°C, the 492‐nm OD was measured, and the inhibition was calculated thus: inhibition (%) = (1 – OD_test_/OD_vehicle control_) × 100.

### 2.11 Western blotting analysis

Cells extracts were prepared at 4°C in RIPA buffer (25 mmol/L Tris‐HCl, pH 7.6, 150 mmol/L NaCl, 1% NP‐40, 1% sodium deoxycholate, 0.1% SDS, 1 mmol/L dithiothreitol and Complete Protease Inhibitor Cocktail [Roche]). Western blotting was performed with commercial primary antibodies against these molecules: Bcl‐2 (1:2000; ab32124, Abcam), BAX (1:2000; ab32503, Abcam), MMP‐9 (1:2000; ab38898, Abcam), cyclin D1 (1:2000; ab134175, Abcam), GAPDH (1:2000; ab8245, Abcam), vimentin (1:1000; 5741T, CST) and E‐cadherin (1:1000; 3195T, CST). The immunoreactive bands were detected using HRP‐conjugated secondary antibodies: goat anti‐rabbit (1:1000, A0208) and goat anti‐mouse (1:1000, A0216), from Beyotime.

### 2.12 Tumorigenesis and staining

Transfected SCC15 cells (2 × 10^7^ cells in 100 μl) were injected into 4‐week‐old Balb/c athymic nude mice (Slake Jingda Experimental Animal Co. Ltd., Hunan, China). The tumour volume, measured weekly, was calculated using the formula: V = πAB^2^/6 (A: the largest diameter; B: the perpendicular diameter). After 5 weeks, the nude mice were killed and weighed. The tumours were removed, fixed with formalin and prepared for generating paraffin‐embedded sections. The paraffin‐embedded tumour sections were stained with H&E or antibodies against vimentin and E‐cadherin, according to the routine IHC method.

### 2.13 Image processing and statistical analysis

All images shown are wide‐field microscopy images that were acquired at sufficient resolution. Results in graphs are shown as means ± SEM from three independent experiments. All statistical data were analysed using SPSS 17.0 software (SPSS, Chicago, IL). Two‐tailed Student's *t* test was used to determine *P* values; *P* < 0.05 was considered significant.

## 3 RESULTS

### 3.1 HULC is highly expressed in OSCC

**Figure 1:**
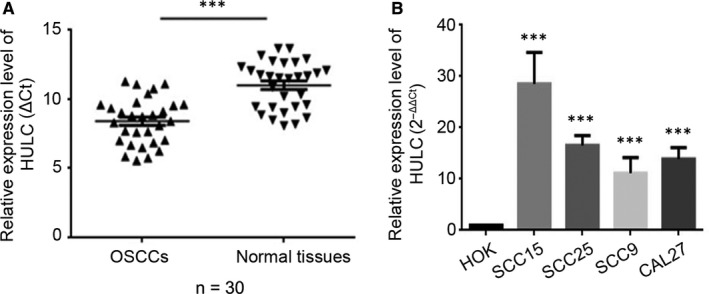
Highly up‐regulated in liver cancer (HULC) is up‐regulated in OSCC tissue specimens and cell lines. (A, B) qRT‐PCR was used to determine the HULC expression level in (A) tumor‐tissue specimens from patients with OSCC, relative to that in adjacent normal tissues (n = 30); and (B) HOK, SCC15, SCC25, SCC9, and CAL27 cell lines. Data are presented as means ± SEM of 3 independent experiments. Student's *t* test, ***P < 0.001

Highly up‐regulated in liver cancer (HULC) mRNA levels were determined using qRT‐PCR. Analysis of 30 pairs of clinical oral cancer tissues and their adjacent normal tissues revealed that HULC expression was higher in the cancer tissues than in the normal tissues (Figure 1A). The clinicopathological features of the 30 OSCC patients are shown in Table 1. We also measured HULC levels in four OSCC cell lines (SCC15, SCC25, SCC9 and CAL27), which revealed that HULC expression was markedly up‐regulated in the cancer cell lines relative to that in a normal oral keratinocyte cell line (human oral keratinocyte (HOK) cells) (Figure 1B). Because similar results were obtained with both the oral cancer tissues and the OSCC cell lines, we used the OSCC cell lines SCC15 and SCC25 for subsequent studies.

**Table 1:** Clinicopathological features of oral squamous cell carcinoma (OSCC) patients

Features	No. of patients	*P* value
Sex		
Male	21	0.0525
Female	9	
Age, y		
<55	17	0.9143
≥55	13	
Tumor size, cm		
<5	22	0.8967
≥5	8	
TNM stage		
I + II	15	0.0006
III + IV	15	
Lymph node metastasis		
Yes	10	0.6322
No	20	

### 3.2 Suppression of HULC reduces proliferation and promotes apoptosis in OSCC cells

**Figure 2:**
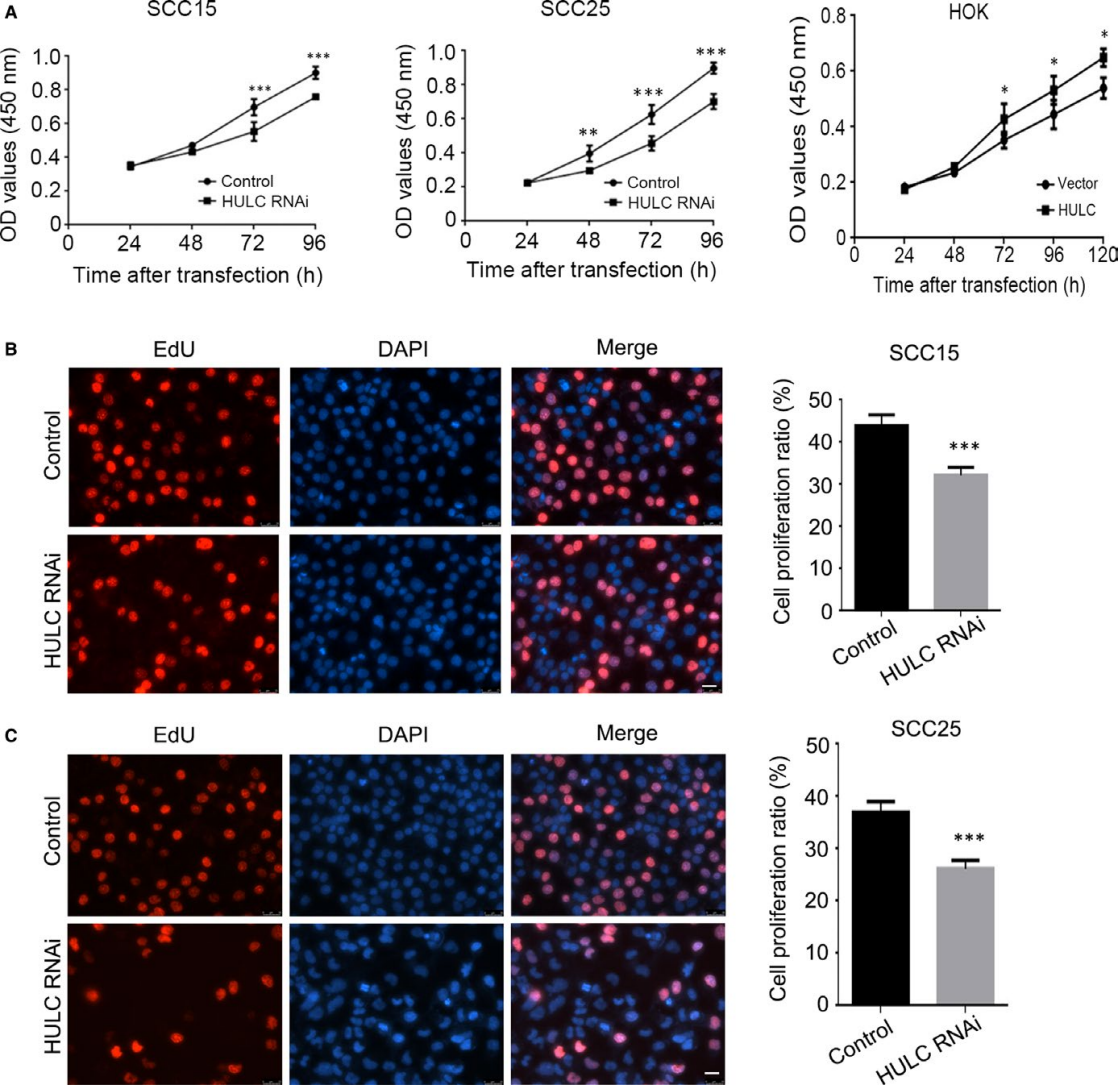
Suppression of HULC expression inhibits OSCC cell proliferation. A, SCC15 and SCC25 cells were transfected with control or HULC siRNA, and the CCK‐8 assay was used to measure cell proliferation after different transfection durations. HOK cells were transfected with vector control or HULC, respectively. The cell proliferation were measured using CCK‐8 assay. (B, C) EdU incorporation assay was used to measure the proliferation ratio of control and HULC‐depleted cells. Data are presented as means ± SEM of three independent experiments. Student's *t *test, *P < 0.05, **P < 0.01, ***P < 0.001; scale bar = 20 μm

To investigate the role of HULC in regulating cell‐proliferation activity, we performed the CCK‐8 assay on SCC15 and SCC25 cells in which HULC was knocked down. Transfection of HULC siRNA into SCC15 and SCC25 cells led to HULC knockdown with an efficiency of roughly 90% and 74%, respectively (Figure S1A,B). Measurement of the 450‐nm absorbance (optical density; OD) at different time‐points revealed that with an increase in transfection time, the proliferation rate of HULC‐depleted cells showed a significant decrease relative to that of control cells (Figure 2A). We also tested the proliferation ratio in HOK cells overexpressed HULC. The up‐regulation of HULC in HOK results in an increase of proliferation rate (Figure 2A). Another assay that involved EdU staining was also performed to confirm the proliferation results; here, nuclei were stained red when the cells were in S phase. Determination of the proliferation ratio in SCC15 and SCC25 cells revealed that after HULC depletion, the ratio was decreased by approximately 12% relative to that in the control group (Figure 2B,C).

**Figure 3:**
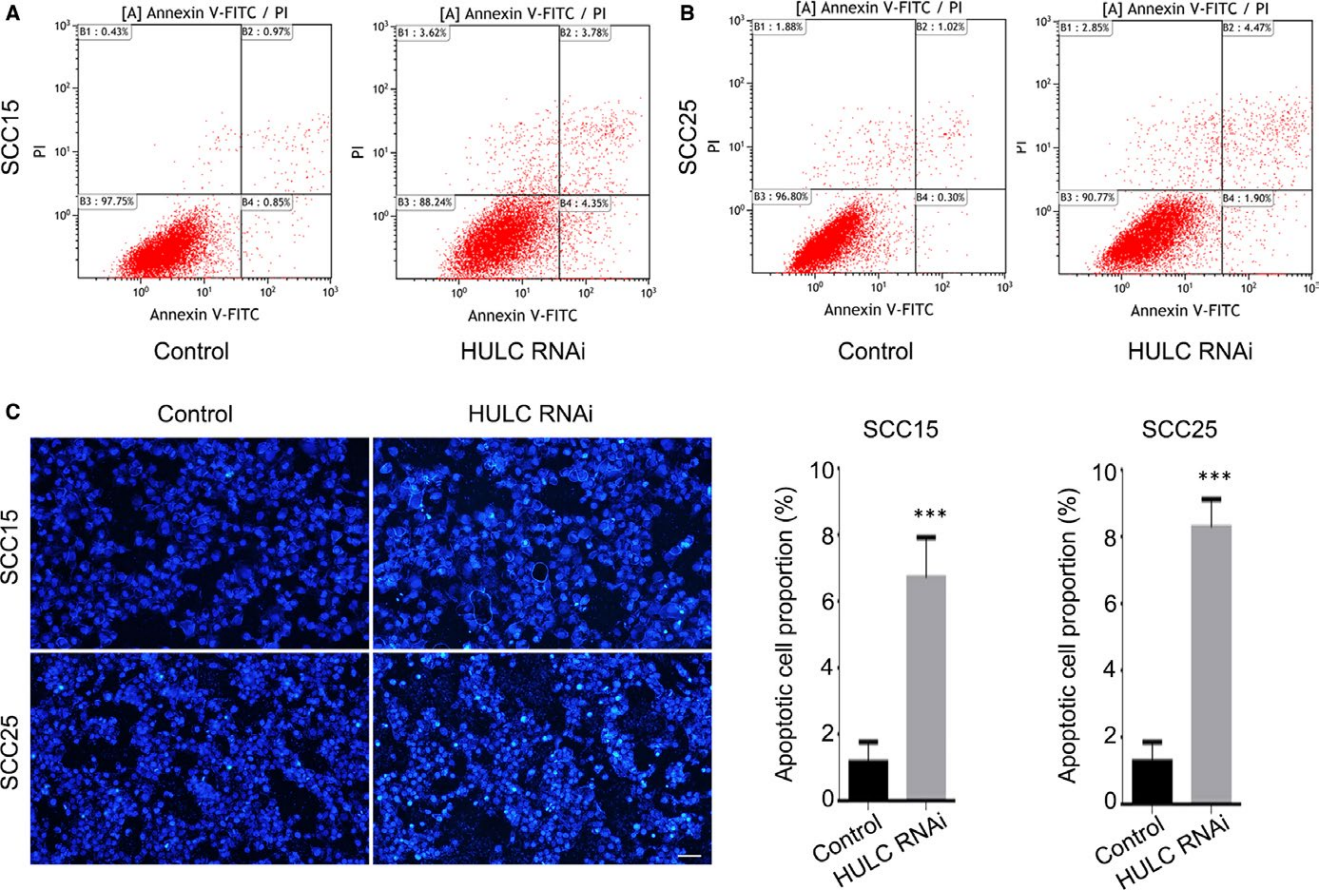
Highly up‐regulated in liver cancer (HULC) depletion increases apoptosis rate of OSCC cells. SCC15 (A) and SCC25 (B) cells were transfected with control or HULC siRNA and then analyzed using flow cytometry. C, Hoechst staining was performed on SCC15 and SCC25 cells transfected with control or HULC siRNA. The proportion of apoptotic cells was quantified. Data are presented as means ± SEM of 3 independent experiments. Student's *t *test, ***P < 0.001; scale bar = 20 μm

Next, the apoptosis rate in HULC‐depleted cells was estimated by performing Annexin V‐FITC/PI dual‐label flow cytometry experiments. In the case of SCC15 cells, the early apoptosis and late apoptosis proportions were 0.85% and 0.97% in the control group, respectively, which were lower than those in the HULC‐depletion group (early apoptosis: 4.35%; late apoptosis: 3.78%; Figure 3A). For SCC25 cells, the early and late apoptosis proportions measured were the following (respectively): HULC‐depletion group, 1.90% and 4.47%; control group, 0.30% and 1.02% (Figure 3B). These results indicate that the suppression of HULC expression strongly promoted apoptosis in SCC15 and SCC25 cells. Here, we also performed Hoechst staining on the SCC15 and SCC25 cells transfected with HULC siRNA and then counted the apoptotic cells in each group: the numbers of apoptotic cells in the HULC‐depletion groups were 5.6‐fold (SCC15) and 7‐fold (SCC25) higher than those in the corresponding control groups, respectively (Figure 3C). Collectively, these findings indicate that HULC depletion reduces the proliferation of OSCC cells and promotes their apoptosis.

### 3.3 HULC down‐regulation inhibits OSCC cell migration and invasion abilities

**Figure 4:**
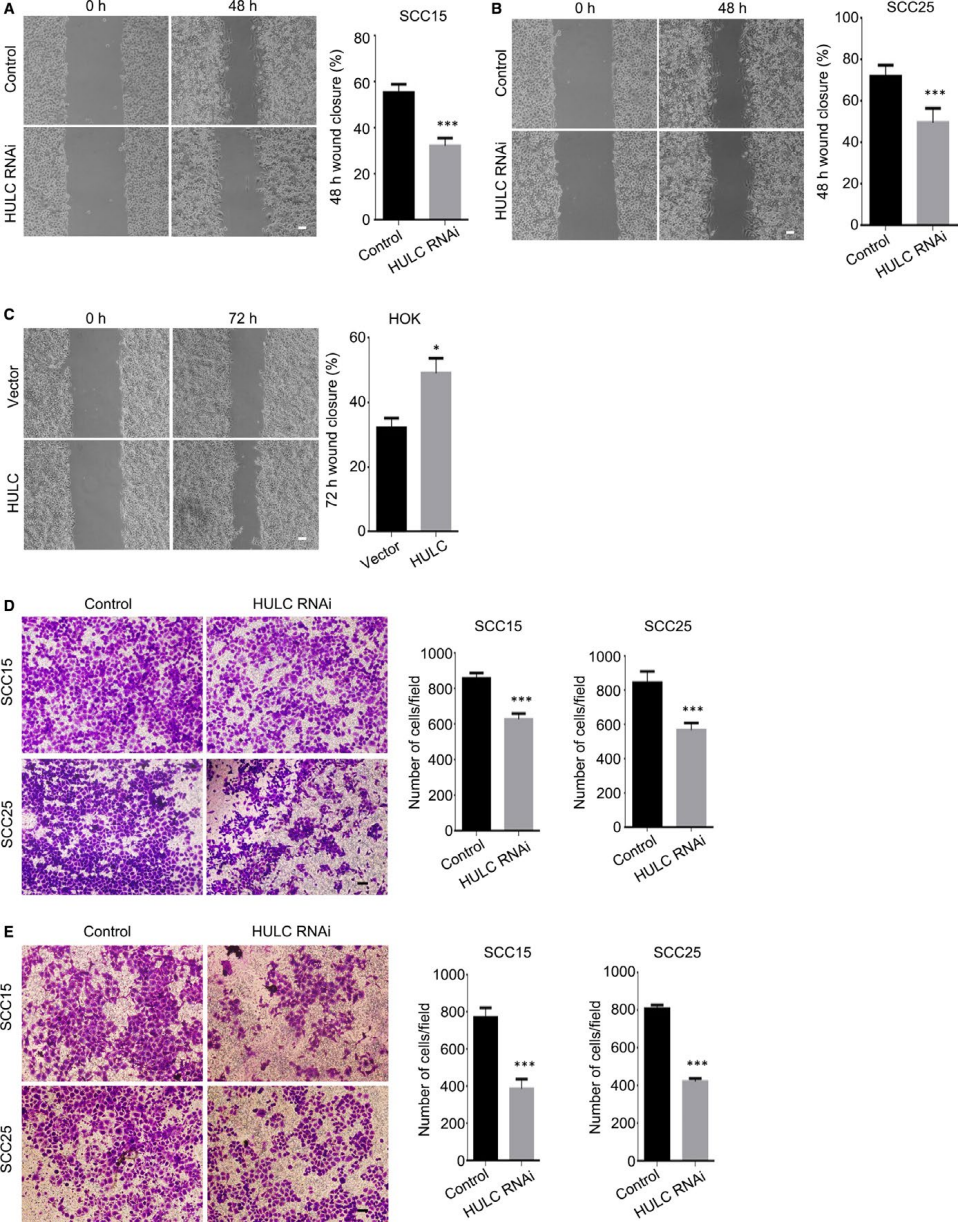
Down‐regulation of HULC inhibits OSCC cell migration and invasion. (A, B) Wound‐healing assays were performed on SCC15 (A) and SCC25 (B) cells transfected with control or HULC siRNA. The scratch area was measured at 0 and 48 h, and the percentage of closure at 48 h was calculated. C, HOK cells stably express HULC were used in wound‐healing assay. The scratch area was measured at 0 and 72 h, and the percentage of closure at 72 h was calculated. (D, E) Transwell assay was performed to quantify the migration and invasion ability of SCC15 and SCC25 cells transfected with control or HULC siRNA. D, After 48‐h transfection, cells were seeded into the upper Transwell chamber (uncoated), and the cells that crossed to the lower chamber were imaged and quantified. (E) After 60‐h transfection, cells were seeded into the upper, Matrigel‐coated Transwell chamber, and the cells that went across the coated chamber were imaged and quantified. Data are presented as means ± SEM of three independent experiments. Student's *t *test, *P < 0.05, ***P < 0.001; scale bar = 20 μm

To determine whether HULC influences OSCC cell migration, we performed wound‐healing assays on control and HULC‐depleted SCC15 and SCC25 cells. Measurement of the scratch area at 0 and 48 hours after wounding revealed that the wound‐closure rate in HULC‐depleted cells was significantly lower than that in control cells (Figure 4A, B). The closure percentages at 48 hours in HULC‐depleted cells were roughly 21% lower (SCC15; Figure 4A) and 25% lower (SCC25; Figure 4B) than those in the control cells. In addition, we also tested the migration ability in HOK cells with high expression of HULC. Up‐regulation of HULC caused higher wound‐closure rate in HOK cells (Figure 4C). The closure percentages at 72 hours in HULC‐overexpressed cells were roughly 16% higher than those in the control cells (Figure 4C). To quantify the migration ability of HULC‐depleted OSCC cells, Transwell assays were used. After 48‐hours incubation, the numbers of HULC‐depleted SCC15 and SCC25 cells that had passed through to the lower chamber were approximately 600 and 580, respectively, which were considerably lower than that in the case of control cells (roughly 820; Figure 4D). Overall, the results suggested that the OSCC cell migration ability was impaired following the depletion of HULC. To investigate the OSCC cell invasion ability, we again used the Transwell invasion assay and counted the cells that had crossed through the chamber coated with Matrigel. HULC depletion strongly suppressed the invasiveness of SCC15 and SCC25 cells, with the invading HULC‐depleted cells in both cases being only around half as many as the invading control cells (Figure 4E). Taken together, these results suggest that HULC regulates OSCC cell migration and invasion.

### 3.4 Drug tolerance of OSCC cells is HULC‐dependent

**Figure 5:**
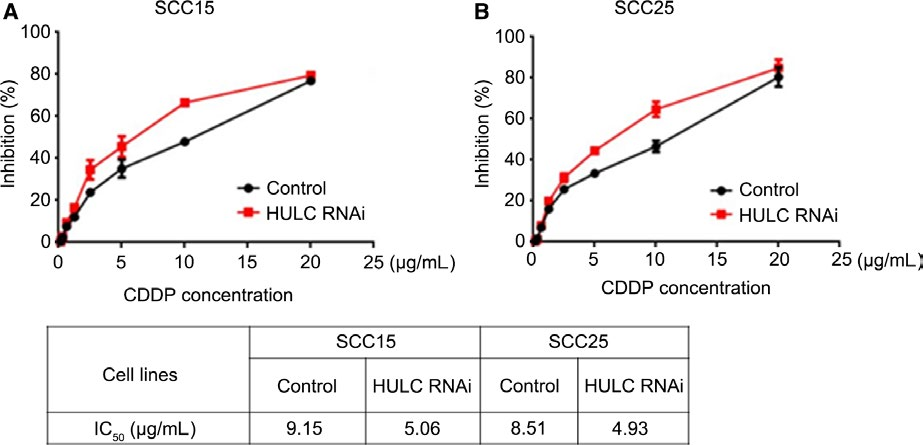
HULC suppression reduces drug tolerance in OSCC cells. (A, B) SCC15 and SCC25 cells transfected with control and HULC siRNA showed a decrease in viability with an increase in CDDP dosage. Inhibition percentages are shown as means ± SEM of 3 independent experiments. C, IC_50_ values obtained with SCC15 and SCC25 cells transfected with control or HULC siRNA

The drug most commonly used for OSCC treatment is cis‐diamminedichloridoplatinum (II) (CDDP).^22^ Thus, we conducted CDDP dose‐response assays to investigate the effect of HULC on OSCC cell viability and drug tolerance. SCC15 and SCC25 cells were transfected with HULC siRNA and then cell viability in the presence of CDDP at various concentrations was measured after incubation for 72 hours. Both control and HULC‐depleted OSCC cells exhibited a CDDP dose‐dependent reduction in viability (Figure 5A, B), but, notably, the cell‐inhibition percentage was increased following HULC down‐regulation, which indicated a diminished CDDP tolerance in HULC‐depleted OSCC cells. Moreover, calculation of the IC_50_ after treating OSCC cells with CDDP yielded lower IC_50_ values for HULC‐depleted cells than control cells (Figure 5C). These results indicate that CDDP inhibits OSCC cell viability in a dose‐dependent manner and that HULC depletion reduces the drug tolerance in OSCC cells.

### 3.5 HULC controls OSCC cell EMT process and tumour growth

**Figure 6:**
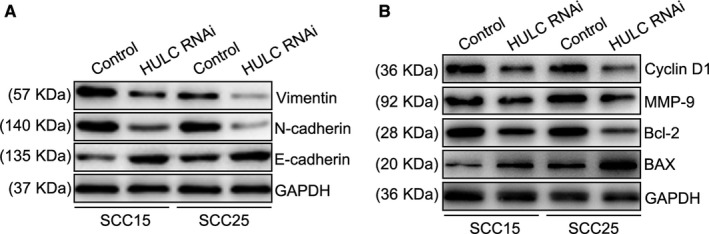
Suppression of HULC affects expression of proteins involved in EMT, cell proliferation, and metastasis. (A, B) SCC15 and SCC25 cells were transfected with control or HULC siRNA, and then cell extracts were immunoblotted with the indicated antibodies

To investigate the molecular basis of HULC regulation in OSCC cells, we examined the expression of several key proteins. Previous studies have shown that EMT is a crucial factor for epithelial cancer metastasis.^23^ Here, to evaluate the EMT process, we suppressed HULC expression and detected EMT markers by performing Western blotting. As compared to control OSCC cells, HULC‐depleted cells showed decreased expression of vimentin and N‐cadherin and increased expression of E‐cadherin (Figure 6A), which indicates that HULC functions in the EMT process in OSCC cells. We next immunoblotted for the following proteins: Bcl‐2 and BAX, which are critical indicators used for detecting cell proliferation and apoptosis^24^, ^25^; MMP‐9, which plays a crucial role in tumour invasion and metastasis^26^; and cyclin D1, a key regulator of cell cycle transition from G1 to S phase.^27^ Our results showed that after HULC knockdown, BAX was up‐regulated, whereas Bcl‐2, MMP‐9, and cyclin D1 were down‐regulated (Figure 6B). These results indicate that HULC participates in the EMT process and affects the expression levels of proteins that are crucial for cell proliferation and invasion.

**Figure 7:**
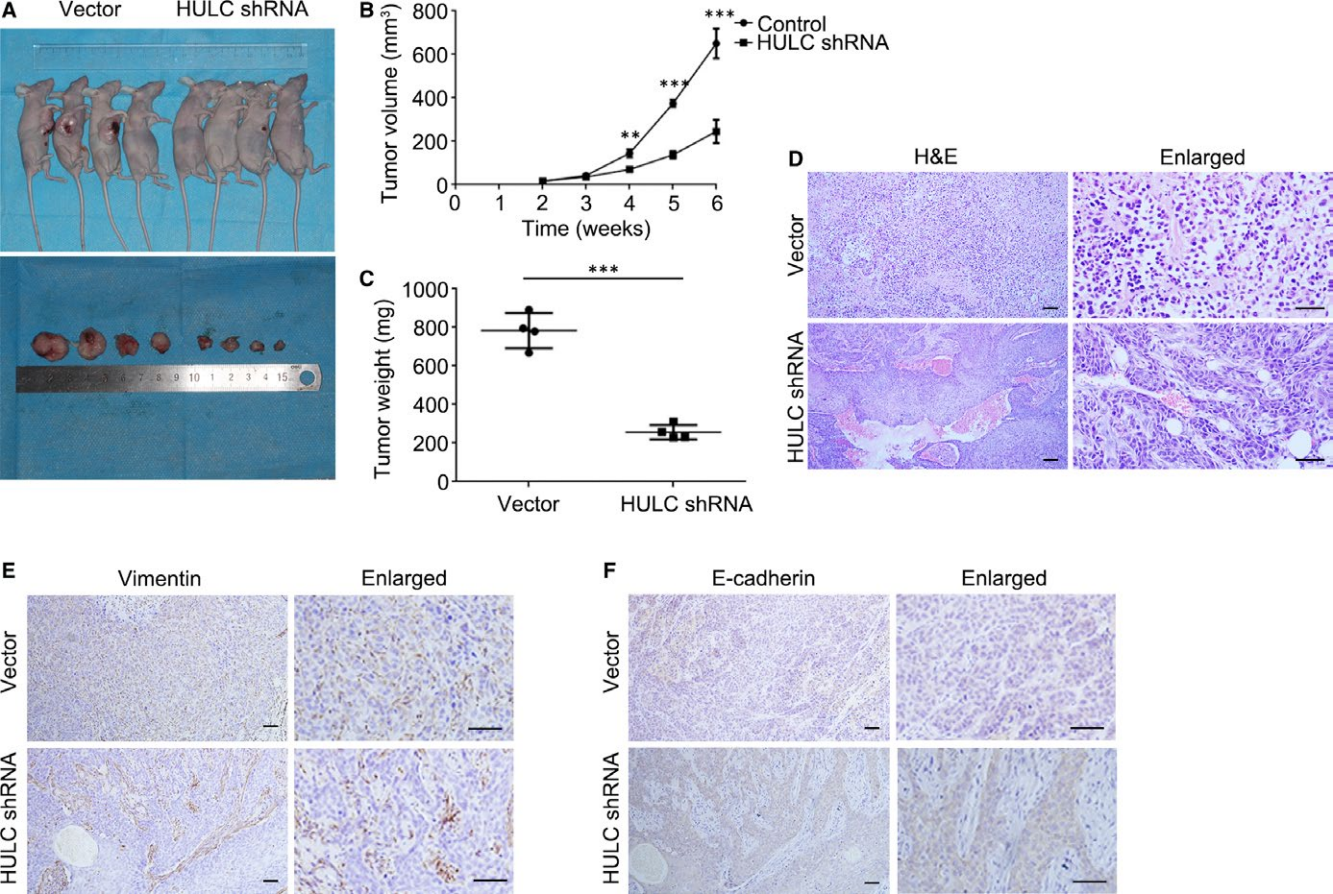
HULC down‐regulation inhibits tumorigenesis. A, Pictures of nude mice bearing xenograft tumors generated using SCC15 stable cells and of surgically removed tumors. B, Tumor growth curve showing change in tumor volume against weeks after injection. Data are presented as means ± SEM of 3 independent experiments. Student's *t *test, **P < 0.01, ***P < 0.001. C, Tumor weight plots of vector‐control and HULC‐depleted groups. Student's *t *test, ***P < 0.001. D, Representative H&E staining and enlarged images from vector‐control and HULC‐depleted tumor groups. E, Images of IHC staining performed for detecting the expression of vimentin and E‐cadherin in vector‐control and HULC‐depleted tumor groups; scale bar = 50 μm

Lastly, to investigate the potential of HULC as a new OSCC therapeutic target, we established a xenograft tumour model by using the SCC15 cell line in nude mice. In SCC15 cells, HULC was knocked down by using a lentiviral vector carrying a GFP‐tagged shRNA (Figure S2A); in the stably transfected SCC15 cells, HULC expression was lowered to approximately 20% of the control level (Figure S2B). All mice developed tumours at the injection sites, but the tumours in the HULC‐depletion group were considerably smaller than those in the empty‐vector group (Figure 7A). Measurement of the tumour growth curve and final weight in the nude mice further revealed that both were suppressed in the HULC‐depletion group relative to control (Figure 7B, C). Moreover, the results of haematoxylin and eosin (H&E) staining showed that SCC15 in the vector group presented poorly differentiated morphology, predominantly immature cells, and a large number of abnormal nuclear divisions, keratinization and almost no visible intercellular bridges. It indicates that the tumour cells are highly malignant. However, in the HULC shRNA group, it exhibited a moderately differentiated morphology, with clear nuclear polymorphisms and nuclear fissures, keratinization, and intercellular bridges, which suggests that the tumour cells are less malignant (Figure 7D). We also did the immunohistochemical (IHC) staining of xenograft tumours (Figure 7E,F), which showed that the EMT maker vimentin was down‐regulated (Figure 7E) in HULC‐depleted tumours but E‐cadherin was up‐regulated (Figure 7F), which agreed with the Western blotting results. Collectively, these results showed that HULC is crucial for tumour growth and promotes the EMT process. Our findings further indicate that HULC could potentially serve as a new therapeutic target in OSCC treatment.

## 4 DISCUSSION

Oral squamous cell carcinoma (OSCC) is the tenth most common malignancy and accounts for 90% of all head and neck malignancies worldwide.^1^ Our study here revealed that HULC expression differed in OSCC specimens and their adjacent non‐tumour tissues, with HULC being up‐regulated in the OSCC tissues. Our results also demonstrated in vitro that HULC down‐regulation can inhibit OSCC cell proliferation, migration, and invasion and induce tumour‐cell apoptosis. Notably, quantification of subcutaneous tumorigenesis in nude mice confirmed that HULC down‐regulation inhibits tumour growth and the EMT process in vivo.

Epithelial‐to‐mesenchymal transition (EMT) is critical for cancer cell invasion and metastasis. This process makes less motile epithelial cancer cells transit into a more motile elongated, fibroblast‐ or mesenchymal‐like phenotype. It is largely attributed to the loss of E‐cadherin which mediates cell‐cell adhesions, along with an increase in matrix‐binding integrins.^28^, ^29^, ^30^, ^31^ Increased expression of basement membrane‐degrading MMPs further contributes to the development of a more invasive mesenchymal‐like phenotype.^32^, ^33^ To confirm the function of lncRNA HULC in OSCC, we examined the expressions of N‐Cadherin, Vimentin and MMP‐9 in response to suppression of HULC. All of them were significantly down‐regulated in SCC15 and SCC25 cells when knockdown HULC. However, HULC depletion increased E‐Cadherin expression in OSCC cells. These results revealed that oral squamous epithelial cells undergo a series of biological changes, including loss of cell‐cell and cell‐basement membrane contact, as well as changes in cell morphology and expression of many proteins. These changes increase the mobility and metastasizing process.

Highly up‐regulated in liver cancer (HULC), originally found in hepatocellular carcinoma and served as an oncogene, has been found dysregulated in various human tumours, such as osteosarcoma, colorectal carcinoma, gastric cancer and diffuse large B‐cell lymphoma.^14^, ^34^, ^35^, ^36^ Although the importance of HULC in regulating cell proliferation, migration and invasion has been demonstrated, the mechanisms underlying HULC overexpression in cancer cells remain unclear. Analysis of the events upstream of HULC expression in hepatocellular carcinoma (HCC) cells has suggested that hepatitis B virus could regulate CREB and thus induce the high expression of HULC.^37^ Moreover, several transcription factors and post‐transcriptional regulators have been shown to up‐regulate HULC expression.^38^ Conversely, studies on the HULC downstream oncogenic pathway have mainly focused on HCC and have reported the up‐regulation or down‐regulation of several proteins by HULC and either gain or loss of function in the tumorigenesis process.^39^ In liver cancer, the lncRNA HULC was reported to bind miRNA‐372 and act as a miRNA sponge regulating the expression of protein kinase cAMP activated catalytic subunit beta (PRKACB). PRKACB plays an important role in the cAMP/PKA signal transduction pathway, which affects a number of cellular processes such as cell proliferation and differentiation.^40^ Moreover, HULC expression was increased in Triple‐negative Breast Cancer（TNBC）tissues and cell lines, which is associated with malignant status and poor prognosis of TNBC patients. While silencing TNBC expression effectively suppressed metastasis through MMP‐2 and MMP‐9 in TNBC cells. It suggested that HULC acts as an independent poor prognostic factor in TNBC patients.^41^ In glioma patient tissues, HULC expression were positively correlated with grade dependency. Silencing HULC suppressed angiogenesis by inhibiting glioma cells proliferation and invasion. This process arrests the cell cycle at G1/S phase via the PI3K/Akt/mTOR signalling pathway. These effects were reversed by overexpression of endothelial cell specific molecule 1 (ESM‐1), which suggests a regulatory role of HULC in the pro‐angiogenesis effect to ESM‐1.^42^ Collectively, HULC is an oncogenic lncRNA and participates in tumour development and progression. Although HULC has been found to be crucial for several cancer types, this is the first report on HULC in human OSCC. We have demonstrated for the first time that HULC is highly up‐regulated in OSCC and is crucial for OSCC cell proliferation, migration and invasion, and our data further suggest that HULC could function as a potential oncogene and promote the malignant progression of OSCC; this provides a basis for the use of HULC as a tumour marker specific for OSCC. However, to further understand HULC regulatory mechanisms in OSCC, the pathways both upstream and downstream of HULC must be investigated in future studies.

In this study, we found that HULC expression was considerably higher in oral cancers than in adjacent normal tissues in patients. However, Kaplan‐Meier survival analysis was not completed because the patient number was limited and because of the loss of follow‐up. Moreover, the relationship between the TNM stage and HULC expression level currently remains unclear. To further enhance our understanding of HULC function in OSCC, a more precise and comprehensive analysis of HULC expression in OSCC patients is required.

## CONFLICT OF INTEREST

The authors confirm that there are no conflicts of interest.

## AUTHOR CONTRIBUTIONS

W. S. and J. T. performed the experiments. Y. W. and Y. S. designed the experiments. S. S. performed the qRT‐PCR analysis. H. Y. and Y. S. wrote the manuscript, with input from all authors.
