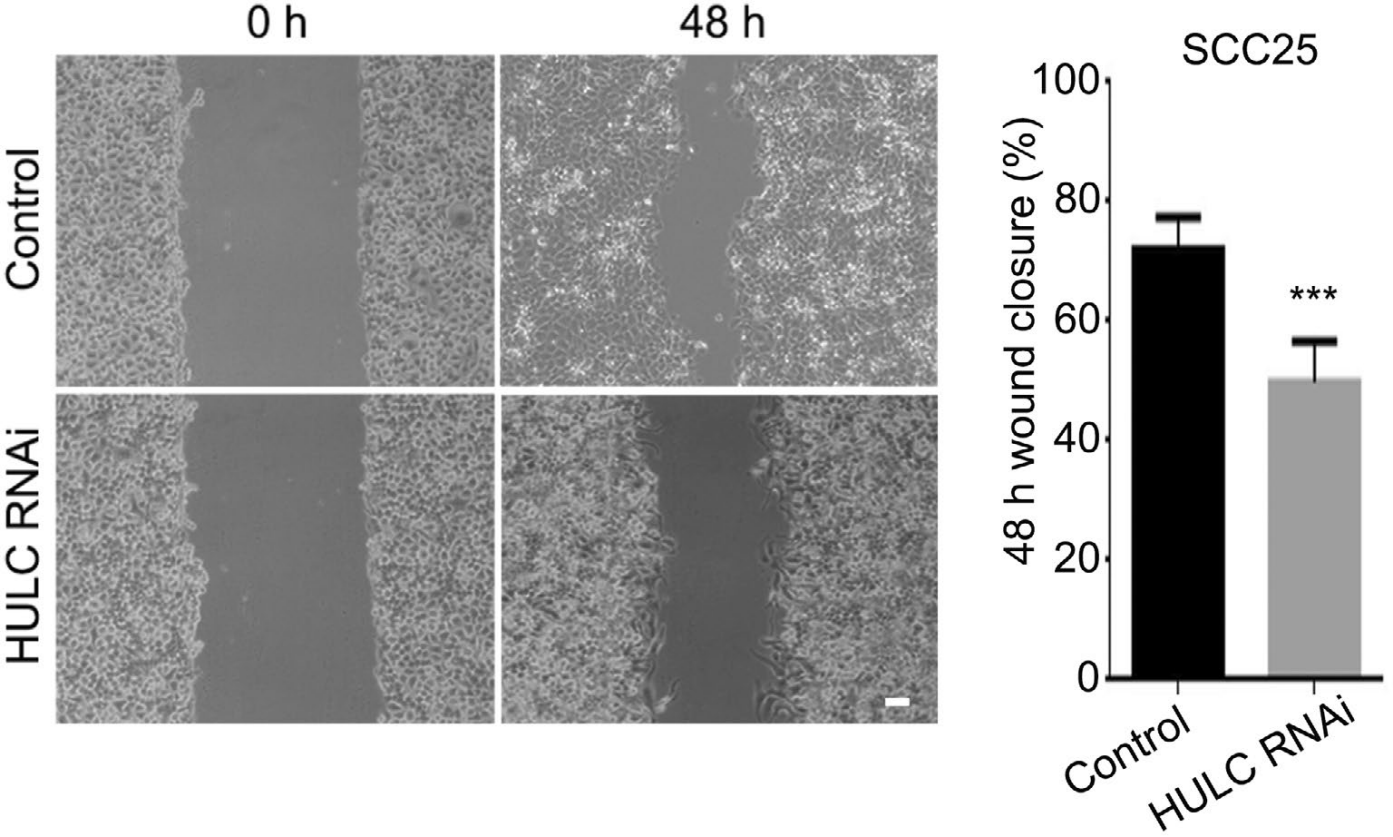# Correction to ‘Long Noncoding RNA Highly Upregulated in Liver Cancer Promotes Epithelial‐to‐Mesenchymal Transition Process in Oral Squamous Cell Carcinoma’

**DOI:** 10.1111/jcmm.71022

**Published:** 2026-01-21

Su W, Tang J, Wang Y, Sun S, Shen Y, Yang H. “Long Non‐Coding RNA Highly Up‐Regulated in Liver Cancer Promotes Epithelial‐to‐Mesenchymal Transition Process in Oral Squamous Cell Carcinoma,” *Journal of Cellular and Molecular Medicine* (2019) 23(4):2645–2655, https://doi.org/10.1111/jcmm.14160.

In the article, there was an error in Figure 4B. The 48‐h control group image of SCC25 was incorrectly placed during manuscript preparation. The correct Figure 4B is shown below. We sincerely apologise for this error.