# Supplementary figures and images for: Long non‐coding RNA highly up‐regulated in liver cancer promotes epithelial‐to‐mesenchymal transition process in oral squamous cell carcinoma
Source: J Cell Mol Med. 2019 Jan 24;23(4):2645–55. doi: 10.1111/jcmm.14160 (PMC6433680; doi:10.1111/jcmm.14160)

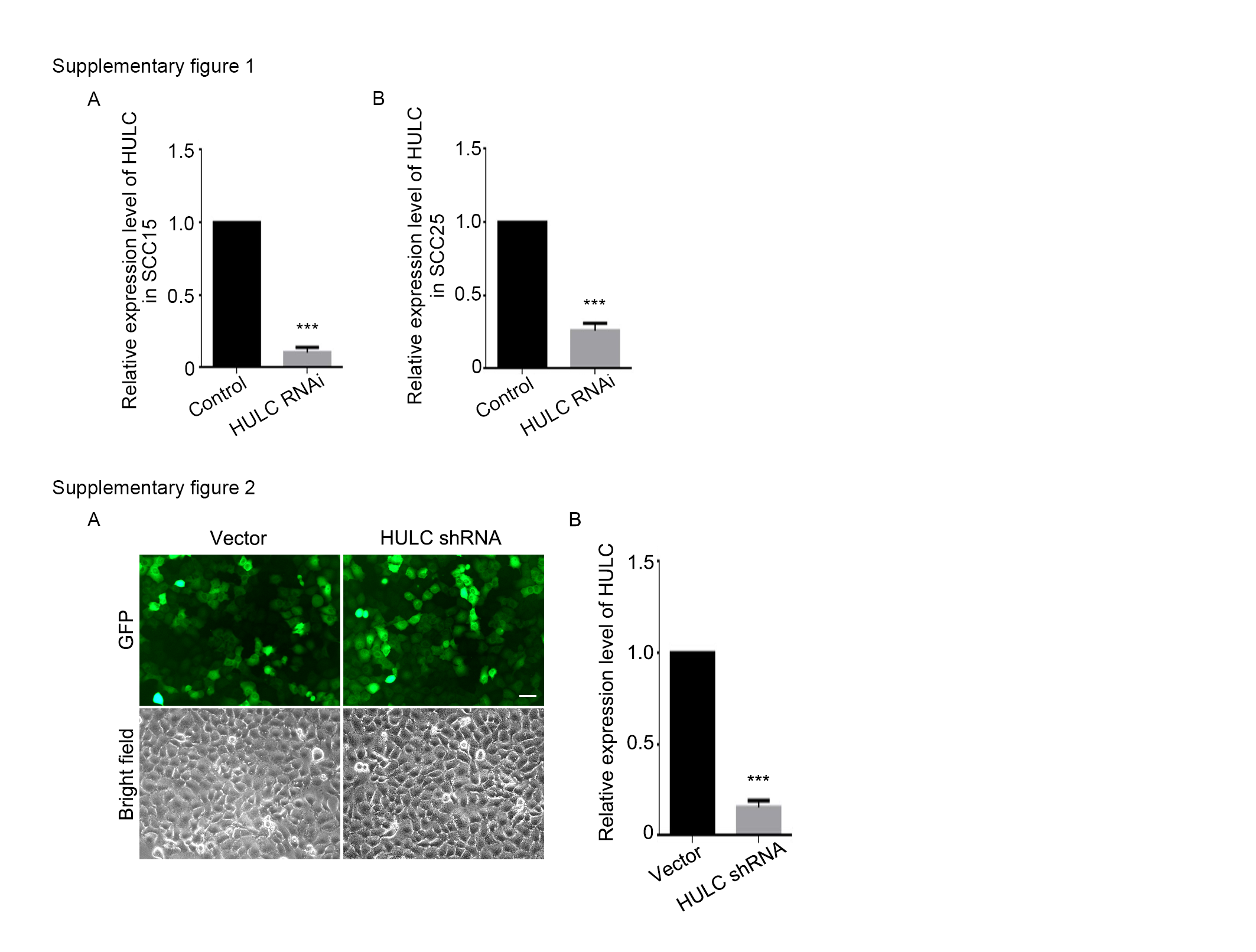

Supplement: Supplementary file 1 [file JCMM-23-2645-s001.tif]
