# Supplementary material for: Long non‐coding RNA highly up‐regulated in liver cancer promotes epithelial‐to‐mesenchymal transition process in oral squamous cell carcinoma
Source: J Cell Mol Med. 2019 Jan 24;23(4):2645–55. doi: 10.1111/jcmm.14160 (PMC6433680; doi:10.1111/jcmm.14160)
Supplement: Supplementary file 2 [file JCMM-23-2645-s002.docx]

**Supplementary figure legends**

**Supplementary Fig. 1 HULC expression levels in SCC15 and SCC25 cells after siRNA-mediated knockdown.** (A, B) qRT-PCR quantification of HULC levels in SCC15 and SCC25 cells transfected with HULC siRNA. Data are presented as means ± SEM of 3 independent experiments. Student’s *t* test, ****P* < 0.001.

**Supplementary Fig. 2 Construction of SCC15 cells stably expressing HULC at low levels by using lentivirus induction.** (A) GFP expression examined under a fluorescence microscope. (B) HULC expression level in the stable cell line quantified using qRT-PCR. Data are presented as means ± SEM of 3 independent experiments. Student’s *t* test, ****P* < 0.001.
